# Thyroid toxicity in lung cancer patients treated with immune-checkpoint inhibitors: a single-center retrospective analysis

**DOI:** 10.3389/fimmu.2026.1757532

**Published:** 2026-04-01

**Authors:** Niccolò Leandro Alessio, Gabriele Ferrari, Antonio Mastrelia, Virginia Valeria Ferretti, Giulia Gambini, Pietro Carlo Lucotti, Francesca Rifaldi, Irene Lanzetta, Anna Tortorella, Sabrina Borgetto, Giulia Galli, Salvatore Corallo, Paolo Pedrazzoli, Francesco Agustoni

**Affiliations:** 1 Department of Internal Medicine and Medical Therapy, University of Pavia, Pavia, Italy; 2 Department of Oncology, Comprehensive Cancer Center, Istituto di Ricovero e Cura a Carattere Scientifico (IRCCS) Policlinico San Matteo Foundation, Pavia, Italy; 3 Epidemiology and Biostatistics Unit, Oncology Department, Fondazione Istituto di Ricovero e Cura a Carattere Scientifico IRCCS Policlinico San Matteo, Pavia, Italy; 4 Department of Internal Medicine, Fondazione IRCCS Policlinico San Matteo, Pavia, Italy

**Keywords:** immune check inhibitor (ICI), immunotherapy toxicity, lung cancer, thyroid irAEs, treatment response

## Abstract

**Background:**

Lung cancer is the leading cause of cancer-related mortality worldwide. Immune checkpoint inhibitors (ICIs) have radically changed the treatment of lung cancer gradually entering all treatment settings. Alongside their clinical benefits, ICIs are associated with immune-related adverse events (irAEs), among which endocrine toxicities, particularly thyroid dysfunctions, represent some of the most frequent.

**Methods:**

We conducted a retrospective analysis of 420 lung cancer patients referred to the oncology unit of IRCCS Policlinico San Matteo in Pavia, between March 2016 and December 2024. Clinical and treatment-related data were reviewed to identify thyroid irAEs. Comparative analyses between patients with and without thyroid dysfunction were performed using descriptive statistics and survival outcomes.

**Results:**

Among 420 lung cancer patients treated with ICIs, 69 (16.4%) developed thyroid irAEs. Most events occurred in the first 6 months, and the majority were grade 1–2 (G1 31.9%, G2 66.7%, G3 1.4%). Thyroid replacement therapy was required in 65.2%, while steroids were used in 13%.

Male sex was associated with a lower incidence of thyroid irAEs (p 0.050), non-small cell lung cancer (NSCLC) not otherwise specified (NOS) histology was associated with a higher risk (p 0.021). Disease stage and treatment line were not significantly correlated.

Patients experiencing thyroid irAEs were more likely to achieve an objective response (CR/PR) compared with those without (p 0.028). Moreover, patients with PD as best response showed a significantly lower incidence of thyroid irAEs compared to those with SD (p 0.010). Duration of response was significantly longer in patients with thyroid irAEs (median 34 vs 17 months; p 0.047).

Time-dependent Cox models did not demonstrate a significant association between thyroid irAEs and progression-free survival - PFS (HR 1.08, p 0.66) or overall survival – OS (HR 1.02, p 0.89).

**Conclusions:**

The occurrence of thyroid irAEs correlated with better tumor response rates and prolonged duration of response, while not significantly impacting PFS or OS. These findings support the hypothesis that thyroid irAEs may serve as a favorable immunologic and prognostic biomarker in the context of ICI therapy.

## Introduction

Lung cancer remains the leading cause of cancer-related mortality worldwide, accounting for nearly 1.8 million deaths annually (1). In Italy alone, approximately 45,000 new cases were estimated in 2024, making it the second most frequent malignancy in men and the third in women (2). Despite advances in screening, surgical techniques, and systemic treatments, the prognosis for advanced-stage disease remains poor, with 5-year survival rates still below 20% (3).

In recent years, immunotherapy has revolutionized cancer treatment by enhancing the body’s immune response against tumor cells. Immune checkpoint inhibitors (ICIs), targeting programmed cell death protein 1 (PD-1), programmed death ligand 1 (PD-L1), and cytotoxic T-lymphocyte–associated protein 4 (CTLA-4), restore antitumor immune surveillance by blocking inhibitory pathways that allow cancer cells to evade recognition (4). ICIs have been approved for more than 15 tumor types, including lung cancer, melanoma and renal cell carcinoma, and are now administered to over a million patients worldwide each year (5).

In advanced non-small cell lung cancer (NSCLC), ICIs, alone or combined with chemotherapy, have become the cornerstone of treatment, especially in non-oncogene addicted NSCLC, entering all treatment stages, from metastatic to localized and locally-advanced disease.

However, the widespread use of ICIs has introduced a novel spectrum of toxicities termed immune-related adverse events (irAEs). These events arise from excessive immune activation and the breakdown of self-tolerance, leading to inflammation in virtually any organ. The overall incidence of irAEs is estimated at 60–70%, with grade ≥3 toxicities occurring in about 10–15% of patients (6). The most frequently affected organs include the skin (30–40%), gastrointestinal tract (10–20%), and endocrine glands (5–20%). Combination regimens targeting both PD-1/PD-L1 and CTLA-4 pathways are associated with a higher risk of severe toxicities compared with monotherapy (7).

Among endocrine irAEs, thyroid dysfunctions are one of the most common. Their incidence varies between 6% and 15% with PD-1/PD-L1 inhibitors and can reach 20–25% with combined immunotherapy (8). Typically, patients present with a transient thyrotoxic phase, followed by persistent hypothyroidism requiring lifelong hormone replacement therapy.

This pattern reflects the direct immune-mediated mechanism, in which massive T lymphocyte infiltration leads to the loss of tolerance to thyroid antigens, with early release of pre-formed thyroid hormones and subsequent rapid-onset hypothyroidism due to depletion of thyroid resources.

The onset of thyroid toxicity usually occurs within 6–12 weeks of treatment initiation but can develop at any time, even after therapy discontinuation. Interestingly, several studies suggest a possible association between the development of thyroid irAEs and improved oncologic outcomes, supporting the hypothesis that autoimmunity may reflect a more robust immune activation (9, 10).

Given the increasing use of ICIs in patients with lung cancer (both in advanced and early stage), understanding the incidence, timing, and clinical impact of ICI-related thyroid dysfunctions is crucial for optimizing patient management. Regular monitoring of thyroid function tests, particularly thyroid-stimulating hormone (TSH) and free thyroxine (FT4), is recommended throughout immunotherapy.

This study aims to evaluate the prevalence, characteristics, and clinical course of thyroid dysfunctions associated with immunotherapy in patients with advanced-stage lung cancer, and to explore potential correlations with treatment outcomes and patient characteristics.

## Materials and methods

### Study design and patient disposition

This is an observational, retrospective, single-center study conducted at IRCCS Policlinico San Matteo in Pavia, including all consecutive patients who received ICI therapy for lung cancer between March 2016 and December 2024.

Clinical data were collected through a comprehensive review of electronic medical records. Relevant demographic, clinical, pathological, and treatment information were collected. Data entry included patient age, sex, smoking status, tumor histology and stage, ICI type and line of therapy, baseline thyroid function tests, timing and characteristics of thyroid adverse events (AEs), management strategies, and oncologic outcomes. Laboratory data were retrieved from the institutional electronic system, and survival outcomes were verified through clinical follow-up records.

Patients needed to have istologically or cytologically confirmed stage III-IV lung cancer, treated with at least one dose of an ICI (anti–PD-1, anti–PD-L1, anti–CTLA-4, or combinations) administered at our institution between March 2016 and December 2024.

The study was approved by the local ethics committee, and all procedures were conducted in accordance with the Declaration of Helsinki and institutional regulatory requirements.

### Endpoints and assessments

The primary endpoint was the incidence of thyroid irAEs during ICI treatment.

Secondary endpoints included the association between thyroid AEs and clinical outcomes, specifically tumor response, duration of response (DOR), progression-free survival (PFS), and overall survival (OS), the identification of clinical and pathological factors associated with the occurrence of thyroid AEs and the characterization of timing, severity, and management of thyroid dysfunction induced by ICIs.

Thyroid function was assessed using serum TSH and FT4 measurements performed at baseline and during treatment according to institutional clinical practice. The reference ranges used in our laboratory were 0.4–4.0 mIU/L for TSH and 8–19 ng/L for FT4.

Anti-thyroid antibodies, including anti-thyroid peroxidase antibodies (anti-TPO), anti-thyroglobulin antibodies (anti-Tg), and anti-TSH receptor antibodies (TRAb), were evaluated when clinically indicated. The laboratory reference ranges were <40 IU/mL for anti-TPO, <40 IU/mL for anti-Tg, and <0.55 IU/L for TRAb.

PFS was defined as the time from ICI initiation to documented disease progression or death from any cause. OS was defined as the time from ICI initiation to death from any cause. DOR was measured from the date of first documented partial or complete response until disease progression or death.

AEs description and grading were performed according to the Common Terminology Criteria for Adverse Events (CTCAE, version 5.0).

According to CTCAE v 5.0, grade 1 toxicities are mild and asymptomatic or mildly symptomatic events not requiring intervention; grade 2 events are moderate, may limit instrumental activities of daily living (ADL) and require minimal medical intervention; grade 3 toxicities are severe or medically significant and often require hospitalization or significant therapeutic intervention; grade 4 toxicities are life-threatening and require urgent intervention.

### Statistical analysis

Continuous variables were described using the mean and standard deviation or the median and interquartile range (IQR: 25th–75th percentiles) for skewed distributions, while categorical data were reported as counts and percentages. Comparisons between categorical variables were performed using the chi-square test or Fisher’s exact test, as appropriate.

To investigate clinical and disease-related predictors of thyroid AEs, univariable and multivariable logistic regression models were fitted including age, sex, smoking status, histology, treatment, and best response. Variables with a p-value lower than 0.2 at univariable analysis entered in multivariable model. Results were reported as odds ratios (ORs) with 95% confidence intervals (95% CIs) and p-value.

PFS and OS were estimated using the Kaplan–Meier method. To account for the time-dependent nature of thyroid AEs, survival analyses were performed using time-dependent covariate Cox models and Mantel–Byar tests. All analyses were conducted using Stata software (release 19.5, StataCorp LLC, College Station, TX, USA). A two-tailed p-value < 0.05 was considered statistically significant.

## Results

A total of 420 patients treated with ICIs were included in the analysis. Thyroid irAEs occurred in 69 patients (16.4%), while 349 patients (83.1%) did not develop thyroid toxicity; data were missing for 2 cases. Between these patients, the median age was 68 years (IQR: 61-75). The majority were male (65.2%), current or former smokers (85.5%). Adenocarcinoma was the most frequent histological subtype (59.4%), followed by squamous cell carcinoma (23.2%), not otherwise specified (NOS) histology (11.6%) and small cell lung cancer (5.8%). Most patients started immunotherapy at stage IV disease (72.5%) and in the first line of treatment (60.9%). Pembrolizumab was the most used agent (42%), followed by Nivolumab (18.8%), Durvalumab (13%), Atezolizumab (11.6%), Cemiplimab (4.3%), and other ICIs (10.1%) (**Table 1**).

**Table 1:** Demographic and disease characteristics of the general population compared to patients who developed thyroid irAEs.

Variable	Total	No AE	Thyroid irAE	P-value
	N=420	N=351	N=69	
Age	68.0 (62.0-74.0)	68.0 (62.0-74.0)	68.0 (61.0-75.0)	0.674
Gender				0.099
Females	110 (26.2%)	86 (24.5%)	24 (34.8%)	
Males	310 (73.8%)	265 (75.5%)	45 (65.2%)	
Smoking habit				>0.90
Never smoker	59 (14.4%)	49 (14.3%)	10 (14.5%)	
Smoker or former smoker	352 (85.6%)	293 (85.7%)	59 (85.5%)	
Diagnosis				0.096
ADK	269 (64.0%)	228 (65.0%)	41 (59.4%)	
SCC	95 (22.6%)	79 (22.5%)	16 (23.2%)	
NOS	22 ( 5.2%)	14 ( 4.0%)	8 (11.6%)	
SCLC	34 ( 8.1%)	30 ( 8.5%)	4 ( 5.8%)	
Disease stage				0.103
III	85 (20.2%)	66 (18.8%)	19 (27.5%)	
IV	335 (79.8%)	285 (81.2%)	50 (72.5%)	
Treatment type				0.598
CT-IO	111 (26.4%)	95 (27.1%)	16 (23.2%)	
Mono-IO	276 (65.7%)	230 (65.5%)	46 (66.7%)	
CT-IO-IO	33 ( 7.9%)	26 ( 7.4%)	7 (10.1%)	
Treatment line				0.348
I	247 (58.8%)	205 (58.4%)	42 (60.9%)	
II	136 (32.4%)	118 (33.6%)	18 (26.1%)	
Adjuvant	1 ( 0.2%)	1 ( 0.3%)	0 ( 0.0%)	
Post-CRT Maintenance	36 ( 8.6%)	27 ( 7.7%)	9 (13.0%)	
ICI				0.508
Pembrolizumab	162 (38.6%)	133 (37.9%)	29 (42.0%)	
Nivolumab	96 (22.9%)	83 (23.6%)	13 (18.8%)	
Atezolizumab	72 (17.1%)	64 (18.2%)	8 (11.6%)	
Durvalumab	39 ( 9.3%)	30 ( 8.5%)	9 (13.0%)	
Cemiplimab	17 ( 4.0%)	14 ( 4.0%)	3 ( 4.3%)	
Nivolumab + Ipilimumab	34 ( 8.1%)	27 ( 7.7%)	7 (10.1%)	

Data are presented as median (IQR) for continuous measures, and n (%) for categorical measures.

irAEs, immune-related adverse events; ADK; adenocarcinoma; SCC, squamous cell carcinoma; NOS, not otherwise specified histology; SCLC, small cell lung cancer; IO; immunotherapy, CT-IO, chemotherapy + immunotherapy, CT-IO-IO, chemotherapy + immunotherapy doublet; CRT, chemo-radiotherapy; ICI, immune-checkpoint inhibitors

Baseline TSH and FT4 values were available for all 420 patients. Normal TSH levels were observed in 366 patients (87.1%), while 54 (12.9%) had abnormal TSH values. Baseline FT4 levels were within the normal range in 408 patients (97.1%) and abnormal in 12 patients (2.9%).

According to CTCAE v 5.0, most thyroid irAEs were grade 2 (46/420, 11%), followed by grade 1 (22/420, 5.2%), and only one case (0.2%) was grade 3. No grade 4–5 events were observed.

Regarding timing, thyroid irAEs most frequently occurred within the first three months of treatment (42/69, 60.9%), with 18 cases within the first month (26.1%), 24 within three months (34.8%), 14 within six months (20.3%), and 13 occurring after six months (18.8%).

Anti-thyroid antibodies were assessed in all 69 patients who developed thyroid irAEs. Among these, anti-TPO antibodies were positive in 19 patients (27.5%), anti-Tg antibodies in 19 (27.5%), and anti-TSH receptor antibodies in 15 (21.7%). Treatment for thyroid toxicity included hormone replacement in 45 of 69 patients (65.2%) and corticosteroids in 9 of 69 patients (13%) (**Table 2**).

**Table 2:** Baseline thyroid function parameters and characteristics of thyroid immune-related adverse events.

Variable	Event (%)
Baseline thyroid function	n = 420
In range TSH Out of range TSH In range FT4 Out of range FT4	366 (87.1) 54 (12.9) 408 (97.1) 12 (2.9)
Any grade thyroid irAEs	n = 69
Grade 1 Grade 2 Grade 3	22 (31.9) 46 (66.7) 1 (1.4)
Onset ≤ 3 months Required thyroid replacement Any auto-antibody detection Anti-TPO Anti-TG Anti-TSHR	42 (60.9) 45 (65.2) 53 (76.8) 19 (27.5) 19 (27.5) 15 (21.7)

TSH, thyroid-stimulating hormone; FT4, free thyroxine 4; irAEs, immune.related adverse events; TPO, thyroid peroxidase; TG; thyroglobulin; TSHR, thyroid-stimulating hormone receptor.

Immunotherapy was temporarily withheld in 23 out of 69 patients (33.3%), with a suspension longer than 6 months in 8 patients (11.6%) often due to overlapping of other irAEs.

Thyroid AEs were slightly more frequent among females (21.8% vs. 14.5% in males; *p* = 0.076). Patients with thyroid AEs achieved higher rates of partial or complete response (CR/PR) compared to those without (15.9% vs. 17.1%, *p* = 0.028). No associations were observed with histology, disease stage, smoking status, treatment line, or ICI type (all p-values higher than 0.096).

Among responders (CR/PR), the median DOR was longer in patients with thyroid AEs compared to those without (mDOR 18 months vs 13 months, *p* = 0.047).

In univariable logistic regression model (**Table 3**), males showed a lower odds of AE respect to females (OR = 0.61 *p* = 0.078), and Stable disease (SD) was associated with higher odds of thyroid AEs compared with disease progression (PD) (OR = 2.21 *p* = 0.010). No association was found with treatment (*p* = 0.643), smoking status (*p* = 0.972) and age (*p* = 0.617). In multivariable logistic model, SD was still associated with higher odds of thyroid AEs compared with disease progression (PD) (OR = 2.21 *p* = 0.011) and males showed a lower odds of AE respect to females (OR = 0.57 *p* = 0.057).

**Table 3:** Crude (Univariable analysis) and adjusted (Multivariable analysis) associations of clinical characteristics and occurrence of thyroid AEs.

Variable	Thyroid AEs Univariable analysis	Thyroid AEs Multivariable analysis
	OR (95%CI) p-value	OR (95%CI) p-value
Gender		
Males vs Females	0.61 (0.35 – 1.06) p=0.078	0.57 (0.32 – 1.02) p=0.057
Age	0.99 (0.96 – 1.02) p=0.617	–
Smoking status		
Yes vs No	0.99 (0.47 – 2.06) p=0.972	–
Hystology		
SCC vs ADK	1.13 (0.60 – 2.12) p=0.712	1.25 (0.65 – 2.39) p=0.502
NOS vs ADK	3.18 (1.25 – 8.05) p=0.015	3.20 (1.23 – 8.33) p=0.017
SCLC vs ADK	0.74 (0.25 – 2.22) p=0.592	0.67 (0.22 – 2.03) p=0.478
Treatment		
IO vs CT-IO	1.19 (0.64 – 2.20) p=0.585	–
CT-IO+IO vs CT-IO	1.60 (0.59 – 4.30) p=0.352	–
Best response		
SD vs PD	2.21 (1.21 – 4.03) p=0.010	2.20 (1.20 – 4.06) p=0.011
CR/PR vs PD	1.39 (0.62 – 3.11) p=0.426	1.54 (0.68 – 3.50) p=0.298

OR, odds ratio; ADK; adenocarcinoma; SCC, squamous cell carcinoma; NOS, not otherwise specified histology; SCLC, small cell lung cancer; IO; immunotherapy, CT-IO, chemotherapy + immunotherapy, CT-IO-IO, chemotherapy + immunotherapy doublet; SD, stable disease; PD, progressive disease; CR, complete response; PR, partial response.

**Figure 1:**
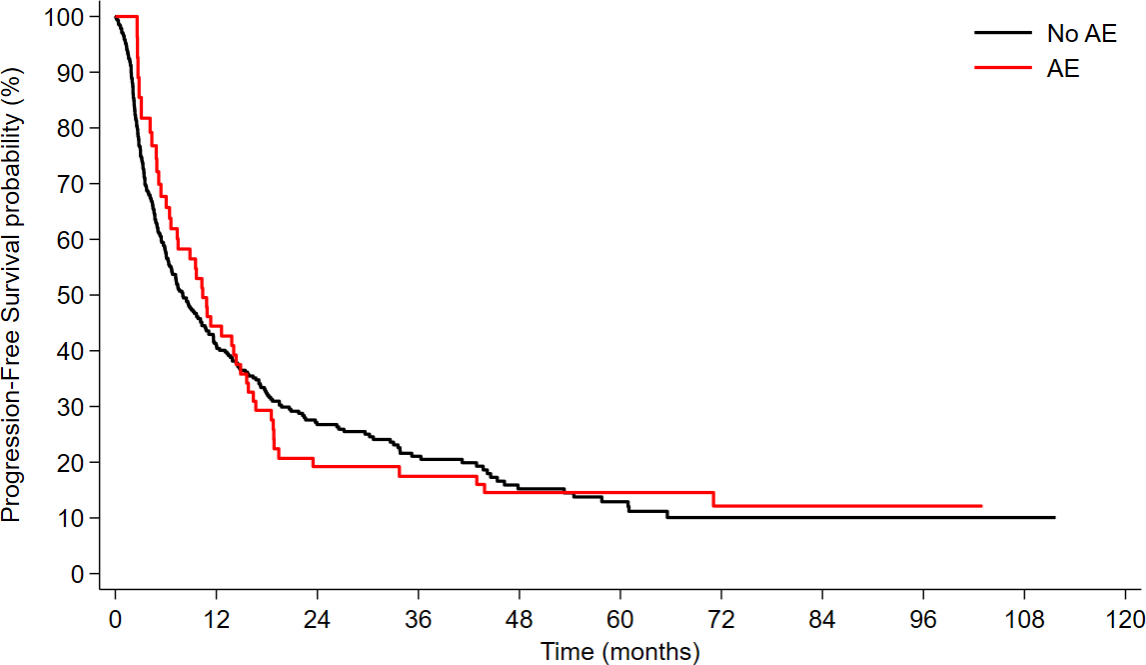
Progression-free survival (PFS) according to the development of immunotherapy-related thyroid toxicity.

In a univariable time-dependent Cox model, the occurrence of thyroid AEs was not associated with PFS (HR 1.08, 95% CI 0.78–1.49, *p* = 0.663) (**Figure 1**). This result was confirmed in multivariable model, after adjusting for smoking status, histology, treatment type and line (HR = 1.09; 95%CI: 0.78-1.51; *p* = 0.623).

**Figure 2:**
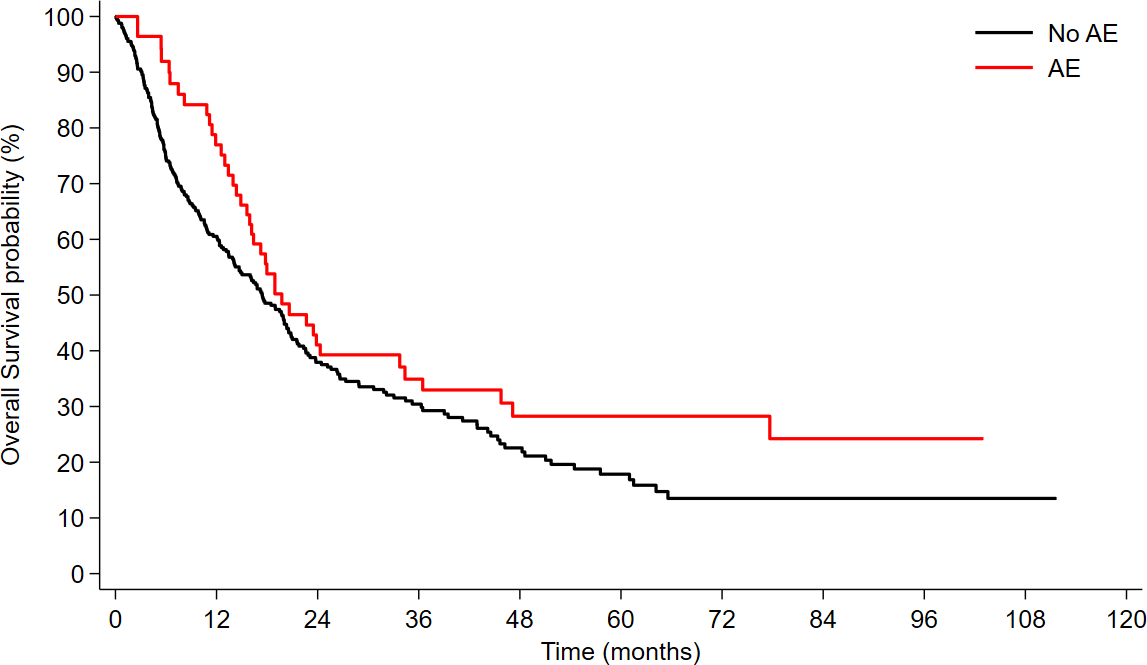
Overall survival (OS) according to the development of immunotherapy-related thyroid toxicity.

Similarly, in the univariable Cox analysis for OS, the development of thyroid AEs was not significantly associated with improved or worsened outcomes (HR 0.81, 95% CI 0.57–1.15, *p* = 0.240) (**Figure 2**). Again, this result was confirmed after adjusting for smoking status, histology, treatment type and line (HR = 0.78; 95%CI: 0.55-1.11; *p* = 0.173).

## Discussion

In this large, single-center cohort of patients with advanced lung cancer treated with immune checkpoint inhibitors, thyroid dysfunction emerged as a relatively frequent immune-related adverse event, occurring in 16.4% of the population. This incidence aligns with previously reported ranges for PD-1/PD-L1 blockade, particularly in real-world settings, where patient heterogeneity and comorbidities may influence toxicity profiles (11–13).

Most thyroid events were low-grade and occurred early, typically within the first three months of therapy, supporting the notion that thyroid dysfunction represents one of the earliest manifestations of immune activation during ICI exposure.

The most common pattern of ICI-related thyroid dysfunction is an initial destructive thyroiditis (thyrotoxic phase) evolving into hypothyroidism, typically within 4–8 weeks (11, 12). Management was generally straightforward, with most patients requiring only levothyroxine supplementation and few needing corticosteroids; nevertheless, one-third experienced temporary interruption of immunotherapy, highlighting the clinical relevance of these events in routine practice.

The pathogenesis likely involves immune activation breaking tolerance toward thyroid antigens, T-cell infiltration of the gland, and local inflammation. In line with the immunobiological framework proposed by recent translational investigations (14), PD-1/PD-L1 blockade may promote expansion of autoreactive T-cell clones, disruption of peripheral tolerance, and possible epitope spreading between tumor and thyroid antigens, ultimately leading to destructive thyroiditis as a byproduct of systemic immune engagement. Some patients have positive anti-thyroid antibodies, but these are neither universally present nor clearly predictive, suggesting that humoral autoimmunity may represent a secondary phenomenon rather than the primary driver of thyroid injury.

In our population antibody positivity was relatively low. This variability indicates that mechanistic pathways may vary among patients, and that standard risk factors (baseline TSH, anti-TPO/Tg positivity) may only partially capture predisposition (13), suggesting a distinct mechanism from classic spontaneous autoimmune thyroiditis.

Our multivariable analysis indicated that male sex was associated with a lower risk of thyroid AEs (*p=*0.050), and that NOS histology conferred higher risk (*p=*0.021). These findings may point toward underlying host or tumor-related factors that predispose to thyroid dysfunction during ICI therapy.

Female sex has been repeatedly associated with a higher incidence of ICI-related thyroid dysfunction in both pooled and single-center series; in recent systematic reviews and large cohort analyses this finding has been attributed to sex-related differences in baseline autoimmunity and immune responsiveness (11, 15).

NOS tumors are characterized by increased tumoral immunogenicity (higher PD-L1 expression, greater T-cell infiltration and clonality, and often higher tumor mutational/neoantigen burdens), features that may predispose to heightened systemic immune activation and thereby to a greater propensity for immune-related toxicities including thyroid irAEs (16, 17).

Nonetheless, the heterogeneity of our population (various histologies, treatment lines, agents) and the modest sample size demand cautious interpretation.

A clinically relevant question is whether thyroid irAEs correlate with improved antitumor activity. We observed an association between thyroid AEs and improved tumor response rates (CR/PR) and longer median duration of response (DOR) among responders (18 vs 13 months, *p=*0.047).

This supports the hypothesis that development of endocrine toxicity may serve as a surrogate marker of enhanced immune activation and hence antitumor efficacy (18).

Indeed, several studies have suggested that ICI-induced endocrinopathies correlate with favourable oncologic outcomes. For example, the study by Street et al. (19) provided a comprehensive evaluation of endocrine irAEs across a large cohort of patients receiving ICIs, demonstrating that the occurrence of endocrine dysfunction—especially thyroid abnormalities—was independently associated with improved PFS and OS; notably, the authors performed time-adjusted analyses to mitigate immortal time bias, reinforcing the robustness of the observed association. In a recent meta-analysis, patients with advanced NSCLC treated with ICIs who developed thyroid irAEs had superior clinical outcomes, including longer PFS (HR 0.54, 95% CI 0.44–0.64) and OS (HR 0.34, 95% CI 0.25–0.44) (20). Another meta-analysis by Cheung et al. (21) further strengthened the evidence supporting thyroid irAes as a potential biomarker of ICIs efficacy. Their analysis demonstrated that patients developing thyroid irAEs experienced significantly improved survival outcomes compared with those who did not, even after adjustment for relevant clinical covariates. Of particular interest, the study characterized the temporal pattern of thyroid dysfunction, observing that most cases occurred early during treatment and frequently followed a biphasic course consistent with destructive thyroiditis. This temporal proximity between treatment initiation and thyroid immune activation supports the hypothesis that thyroid autoimmunity may represent an early pharmacodynamic signal of immune system engagement.

However, in our time-dependent Cox models, the occurrence of thyroid AEs was not significantly associated with either PFS (HR 1.08, 95% CI 0.78–1.49) or OS (HR 1.02, 95% CI 0.74–1.40). The absence of a statistically significant correlation between thyroid irAEs and clinical outcomes in our cohort may be attributable to differences in study design, sample size, heterogeneity of histologies and ICI agents, follow-up durations, and differences in how thyroid toxicity was defined and timed.

Moreover, the relatively low number of survival events observed to date, is likely to reduce the statistical power to detect moderate associations. It is also possible that thyroid AEs, while associated with response, may not consistently translate into long-term survival advantage in unselected real-world populations.

The lack of a clear survival advantage associated with thyroid irAEs in our cohort suggests that, while these events may reflect immune activation, they do not uniformly translate into sustained therapeutic benefit. Alternatively, the prognostic value of thyroid toxicity may be context-dependent, varying according to tumor biology, ICI type, or interaction with other irAEs. Further multicenter studies with standardized endocrine monitoring are needed to clarify these nuances.

From a clinical perspective, the incidence and timing of thyroid AEs underscore the importance of systematic thyroid monitoring in patients undergoing ICI therapy. Guidelines from both the American Society of Clinical Oncology (ASCO) and the European Society of Medical Oncology (ESMO) recommend thyroid function tests every 3–6 weeks during treatment (22, 23).

Early identification of thyroid dysfunction allows timely initiation of hormone replacement and may reduce morbidity without necessarily interrupting immunotherapy. This should always be accompanied by appropriate monitoring of cortisol and ACTH levels, in order to enable early intervention in the event of immune-mediated adrenal insufficiency before introducing thyroid hormone replacement therapy.

In our cohort, immunotherapy suspension occurred in 33.3% of patients with thyroid AEs; however, given the mostly mild nature of these events, it may be appropriate in many cases to continue treatment while managing thyroid dysfunction concurrently (24).

Finally, our findings highlight that the relationship between thyroid AEs and oncologic outcome is complex. The observed improvement in response and DOR suggests that thyroid autoimmunity may be a biomarker of heightened antitumour immunity, but the absence of significant PFS/OS benefit implies that other factors modulate survival. It is also possible that the benefits of thyroid AEs are counterbalanced by other risks or complex interactions (e.g., requiring treatment interruption).

Our study has several limitations. First, its retrospective design may introduce bias (e.g., ascertainment, selection). Second, thyroid monitoring (timing, auto-antibody testing) was not completely uniform across patients. Third, our cohort included diverse tumour histologies and different ICI agents and lines. Fourth, the number of events (especially survival endpoints) may have limited statistical power to detect moderate associations. Finally, we lacked detailed data on immunologic biomarkers. These limitations must be acknowledged when interpreting the findings.

However, its strengths include a large sample size, comprehensive clinical annotation, and the use of robust statistical methods to address time-dependent bias.

In conclusion, thyroid dysfunction is a common, early, and generally manageable irAE in patients with lung cancer undergoing immunotherapy. While associated with higher response rates and prolonged duration of response, thyroid toxicity did not independently predict survival outcomes in time-dependent analyses. These findings reinforce the importance of routine thyroid monitoring and highlight the need for further research to elucidate the biological and prognostic significance of endocrine irAEs in the immunotherapy era. Future prospective studies with uniform definitions and population, longer follow-up, immune-biomarker and cytokine expression profile (ie. IL-1β and IL2) integration are needed to refine the prognostic value of thyroid AEs and to optimize treatment strategies in this setting.

## Data Availability

The raw data supporting the conclusions of this article will be made available by the authors, without undue reservation.
